# Long-term outcomes of psychological treatment for posttraumatic stress disorder: a systematic review and meta-analysis

**DOI:** 10.1017/S003329172100163X

**Published:** 2021-07

**Authors:** Maxi Weber, Sarah Schumacher, Wiebke Hannig, Jürgen Barth, Annett Lotzin, Ingo Schäfer, Thomas Ehring, Birgit Kleim

**Affiliations:** 1 Division of Clinical Psychology and Psychotherapy, Freie Universität Berlin, Berlin, Germany; 2 Division of Clinical Psychological Intervention, Freie Universität Berlin, Berlin, Germany; 3 Department of Psychology, Philips University of Marburg, Marburg, Germany; 4 Clinical Psychology and Psychotherapy, Department of Psychology, Philipps University of Marburg, Marburg, Germany; 5 Institute for Complementary and Integrative Medicine, University Hospital Zurich, Zurich, Switzerland; 6 Department of Psychiatry and Psychotherapy, University Medical Center Hamburg-Eppendorf, Hamburg, Germany; 7 Department of Psychology, LMU Munich, Munich, Germany; 8 Department of Psychology, University of Zurich, Zurich, Switzerland; 9 Department of Psychiatry, Psychotherapy and Psychosomatics, Psychiatric University Hospital Zurich, University of Zurich, Zurich, Switzerland

**Keywords:** Efficacy, long-term, meta-analysis, posttraumatic stress disorder, psychological treatment, PTSD

## Abstract

Several types of psychological treatment for posttraumatic stress disorder (PTSD) are considered well established and effective, but evidence of their long-term efficacy is limited. This systematic review and meta-analysis aimed to investigate the long-term outcomes across psychological treatments for PTSD. MEDLINE, Cochrane Library, PTSDpubs, PsycINFO, PSYNDEX, and related articles were searched for randomized controlled trials with at least 12 months of follow-up. Twenty-two studies (*N* = 2638) met inclusion criteria, and 43 comparisons of cognitive behavioral therapy (CBT) were available at follow-up. Active treatments for PTSD yielded large effect sizes from pretest to follow-up and a small controlled effect size compared with non-directive control groups at follow-up. Trauma-focused treatment (TFT) and non-TFT showed large improvements from pretest to follow-up, and effect sizes did not significantly differ from each other. Active treatments for comorbid depressive symptoms revealed small to medium effect sizes at follow-up, and improved PTSD and depressive symptoms remained stable from treatment end to follow-up. Military personnel, low proportion of female patients, and self-rated PTSD measures were associated with decreased effect sizes for PTSD at follow-up. The findings suggest that CBT for PTSD is efficacious in the long term. Future studies are needed to determine the lasting efficacy of other psychological treatments and to confirm benefits beyond 12-month follow-up.

## Introduction

Posttraumatic stress disorder (PTSD) is a highly prevalent and chronic mental disorder (Kessler et al., 2017), associated with personal (Schnurr, Lunney, Bovin, & Marx, 2009) and societal costs (McGowan, 2019). Half (52%) of individuals with PTSD suffer from co-occurring depression (Rytwinski, Scur, Feeny, & Youngstrom, 2013); a comorbidity associated with severe symptoms, reduced functioning, and poorer treatment response in PTSD (Bedard-Gilligan et al., 2015; Haagen, Heide, Mooren, Knipscheer, & Kleber, 2017). Given its chronic course and high personal and economic burden, it is crucial to identify effective psychological treatments for PTSD both in the immediate and long-term phase.

Numerous psychological PTSD treatments have been developed which can be differentiated by content (e.g. Ehlers et al., 2010). Trauma-focused treatment (TFT) mainly focusses on processing the individual's memory of the trauma and/or its meaning. Trauma-focused cognitive behavioral therapy (TF-CBT) typically incorporate psychoeducation, homework, relaxation, and cognitive and/or behavioral-based components (e.g. cognitive therapy, Ehlers & Clark, 2000; cognitive processing therapy, Resick & Schnicke, 1992; prolonged exposure, Foa & Rothbaum, 1998; Foa, Hembree, & Rothbaum, 2007; narrative exposure therapy, Schauer, Neuner, & Elbert, 2011). Eye Movement Desensitization and Reprocessing (Shapiro, 1995) recalls the traumatic memory using bilateral movements and some core elements of TF-CBT. Non-TFTs typically address coping with symptoms, emotion regulation, or current problems in life without a primary focus on the trauma. Techniques of non-TF-CBT comprise, inter alia, anxiety management, relaxation, stress management, social skills training, positive thinking, assertiveness training, or thought stopping (International Society for Traumatic Stress Studies, 2015; e.g. stress inoculation training, Veronen & Kilpatrick, 1983; seeking safety, Najavits, 2002). Series of other PTSD treatments exist (e.g. psychodynamic therapies or hypnotherapy), but are less frequently studied (Cusack et al., 2016).

Several systematic reviews and meta-analyses demonstrate the efficacy of psychological treatment for PTSD (e.g. Benish, Imel, & Wampold, 2008; Chen, Zhang, Hu, & Liang, 2015; Cusack et al., 2016; Gerger *et al*. 2014; Haagen, Smid, Knipscheer, & Kleber, 2015; Lambert & Alhassoon, 2015; Lenz, Haktanir, & Callender, 2017; Sloan, Gallagher, Feinstein, Gayle Beck, & Keane, 2013; Thompson, Vidgen, & Roberts, 2018; Watts et al., 2013). TF-CBT has the strongest empirical support and current practice guidelines recommend this type of psychological treatment as first-line therapy for PTSD (e.g. American Psychological Association, [APA], 2017; Schäfer et al., 2019). Current evidence for TF-CBT and psychological treatment for PTSD in general, however, mainly relies on short-term outcomes; meta-analyses investigating its long-term benefits (e.g. 12 months after treatment) are largely missing. Understanding the long-term outcomes of psychological treatment for PTSD and identifying moderators of sustainable gains is critical for both clinical practitioners and researchers, particularly to inform clinical decision-making and to stimulate research into effective psychological treatment (e.g. in specific patient groups).

Seven recent systematic reviews and meta-analyses examined the lasting benefits of psychological treatment in adult PTSD. Their findings mainly represent treatment effects at short-term to medium-term, indicating medium to large improvements in PTSD symptoms up to 6 months of follow-up (Bisson, Roberts, Andrew, Cooper, & Lewis, 2013; Carpenter et al., 2018; Ehring et al., 2014; Kline, Cooper, Rytwinksi, & Feeny, 2018; Lee et al., 2016; Mavranezouli et al., 2020; van Dis et al., 2020). Two of these studies give insights into the long-term treatment effects of PTSD, i.e. at 12-month follow-up and above. One meta-analysis investigated evidence-based treatments for PTSD at medium-term, with at least 6 months of follow-up (Kline et al., 2018). The findings were based on uncontrolled comparison only, and showed larger treatment effects for active treatments from baseline to medium-term follow-up (*d* = 2.14) compared to active control conditions for the same period (*d* = 1.04). Uncontrolled comparisons between psychological treatments for PTSD demonstrated no significant differences from pretest to follow-up. For the posttest to follow-up phase, exposure-based treatments were superior, while CBTs without exposure were inferior to all other treatments combined. Of the 32 included trials, eight studies (25%) had a 12-month follow-up. Only one study comprised more than 12 months of follow-up, making it difficult to disentangle any long-term benefit from medium-term outcomes. Another meta-analysis focused on CBT for anxiety disorders compared with usual care or wait-list group with at least 1 month of follow-up (van Dis et al., 2020). Longer-term outcomes for PTSD alone showed medium effects at 6–12 month of follow-up and large treatment effects compared with the control group at more than 12 months of follow-up. The analyses were limited to direct comparisons, and 16 studies with a follow-up of at least 6 months were available for PTSD.

This systematic review and meta-analysis aimed to investigate the long-term outcomes across psychological treatments for adults with PTSD. We aimed to assess PTSD severity and comorbid depressive symptoms at least 12 months after treatment completion. Using comparisons both within and between studies, we aimed to increase the number of available studies with a long-term follow-up but simultaneously control for time and placebo effects. We examined whether (1) psychological treatment differed from control groups and whether (2) TFT differed from non-TFT in PTSD severity and comorbid depressive symptoms at long-term follow-up. In addition, we investigated specific moderators and their potential impact on long-term benefits of psychological treatment for PTSD.

## Method

We followed the Preferred Reporting Items for Systematic Reviews and Meta-Analyses (PRISMA) statement for conducting and reporting this meta-analysis (Liberati et al., 2009, see Appendix A). The study protocol was not registered a priori.

### Eligibility criteria

Studies were selected if they comprised (1) face-to-face psychological treatment for PTSD, (2) adult participants, (3) at least 70% of participants diagnosed with PTSD (e.g. according to DSM-IV/V, ICD-10); (4) either active or passive, nonpharmacological control conditions (e.g. supportive counseling, wait-list) or psychological treatment as comparators; (5) PTSD severity as primary outcome measured at least 12 months after the end of treatment; (6) a randomized controlled trial design, and (7) at least ten participants per treatment arm. Trials were included based on any type of trauma, type of setting (e.g. inpatients, outpatients), presence of comorbidity, or adjuvant drug treatment (e.g. by prescription or as part of the study protocol).

### Selection of studies

A systematic literature search in MEDLINE, PsycINFO, PSYNDEX, PTSDpubs, and Cochrane Library was conducted for articles in English or German language until November 7, 2019. The search strategy derived from the preparation of the German S3 treatment guideline for PTSD (Schäfer et al., 2019), and included the following keywords: (PTSD OR OR Posttraumatische Belastungsstörungen or PTBS) AND ((treatment trial OR randomized controlled trial) or (*indexed by a thesaurus term as a clinical trial*)). In addition, we performed a systematic snowball search by screening reference lists from included primary studies and relevant review articles. Two researchers independently screened articles and decided on eligible studies. In cases of disagreement between the researchers, a third researcher decided on eligibility.

### Data extraction

Several study characteristics were extracted (see Appendix A). Means and standard deviations or reported effect sizes for PTSD severity (primary outcome) and comorbid depressive symptoms (secondary outcome) were extracted at baseline, posttest, and at ⩾12 months after treatment completion. In cases of multiple follow-up intervals, data from the latest was used (e.g. Karyotaki et al., 2016; Kline et al., 2018). Data for clinician-rated PTSD and intent-to-treat samples (ITT) were extracted if available. If further statistical data or data subsets were needed (e.g. for adult subsample), we contacted the study authors and sent a follow-up e-mail in case of non-response (57% response rate). One researcher (MW) extracted data, which were cross-checked by a second (SSch) to ensure accuracy.

### Treatment coding

We coded treatment conditions as psychological treatment or control. Psychological treatment was classified as TFT or non-TFT (Ehring et al., 2014; see Ehlers et al., 2010 for discussion). Control conditions were rated as active, if interventions were not directive or trauma-focused such as supportive counseling or treatment as usual (TAU), and served to control for non-specific mechanisms (e.g. Kline et al., 2018; Lambert & Alhassoon, 2015; Powers, Halpern, Ferenschak, Gillihan, & Foa, 2010). We classified control groups as passive if there was no clinician involvement, i.e. during wait-list. Treatment coding was performed by two independent researchers (MW, WH), and a third (BK) was consulted if raters disagreed.

### Study quality assessment

The included studies were assessed using the six domains from the Cochrane risk of bias tool (Higgins et al., 2011): (1) random sequence generation, (2) allocation concealment, (3) blinding of participants, personnel, and (4) outcome assessment, (5) incomplete outcome data (e.g. if no ITT data were available for follow-up analysis), and (6) selective outcome reporting (e.g. if studies deviated from trial registration). All domains were rated as either low, unclear (unknown), or high risk of bias closely following the recommendations for risk of bias ratings in psychotherapy research (see Munder, & Barth, 2018). Two researchers independently (MW, WH) coded risk of biases and consulted a third (SSch) in case of disagreement.

### Statistical analyses

#### Effect size calculation

Two types of effect sizes were estimated using Hedges’ *g* (Hedges, 1981). Within-group effect sizes were obtained by subtracting the follow-up (or posttest) mean from the baseline (or posttest) mean. For between-group effect sizes, the control group mean was subtracted from the treatment group at follow-up or posttest divided by the pooled standard deviation. For within-group effect sizes, the standard deviation within groups was used including the correlation between the two measurements (Borenstein, Hedges, Higgins, & Rothstein, 2009). Both types of effect sizes used were corrected for small sample biases (Hedges & Olkin, 1985). A magnitude of 0.2, 0.5, and 0.8 represents a small, medium, and large effect size, respectively (Cohen, 1977). Positive effect sizes indicate improved symptoms, while the width of the respective 95% confidence interval (CI) quantifies its precision (Borenstein et al., 2009).

Comprehensive meta-analysis software, version 3 (Biostat) was applied to pool effect sizes using a random-effects model. If no correlation was available to calculate within-group effect sizes, sensitivity analyses were performed by replacing the coefficient with *r* = 0.2, *r* = 0.5 and *r* = 0.8; the default value was set to *r* = 0.5 (*k* = 10; Borenstein et al., 2009). Heterogeneity of effect sizes was tested with the *Q*-statistic, the *I*^2^ value, and by visual inspections of forest plots. A *p*-value of the *Q*-statistic below 0.05 indicates heterogeneity (Cochran, 1954). *I*^2^ values range from 0 to 100% and suggest presence of low (25%), medium (50%), and large (75%) heterogeneity (Higgins & Thompson, 2002).

#### Subgroup analysis

Subgroup analyses of treatment conditions (active treatment *v.* control condition), treatment types (TFT *v.* non-TFT) were performed for the primary and secondary outcome. Dropout rates (i.e. ratio of participants initiating but not completing treatment; Ehring et al., 2014) were calculated for both conditions and treatment subgroups. For PTSD severity, six additional variables were analyzed: proportion of female participants [high (⩾50%) *v.* low (<50%); Sloan et al., 2013; Watts et al., 2013], type of population (military personnel *v.* civilian; Kline et al., 2018), treatment format (individual *v.* group-based; Ehring et al., 2014; Haagen et al., 2015), average number of treatment sessions [high (⩾10 sessions) *v.* low (<10 sessions); Lambert & Alhassoon, 2015], outcome measure (self-rated *v.* clinician-assessed, Lambert & Alhassoon, 2015), and type of analysis at follow-up (Kline et al., 2018). Analyses on within-group effect sizes were conducted using the mixed-effect model and the *Q*-statistics (Borenstein et al., 2009) if at least three comparisons per subgroup were available.

#### Publication bias

We applied Egger's regression test (Egger, Davey Smith, Schneider, & Minder, 1997) and Duval and Tweedie's trim-and-fill procedure (Duval & Tweedie, 2000) to test publication bias if data sets contained at least six studies presenting no substantial heterogeneity (Ioannidis & Trikalinos, 2007; Rothstein, Sutton, & Borenstein, 2005; Sterne, Becker, & Egger, 2006; Terrin, Schmid, Lau, & Olkin, 2003).

## Results

### Included studies

**Fig. 1.**
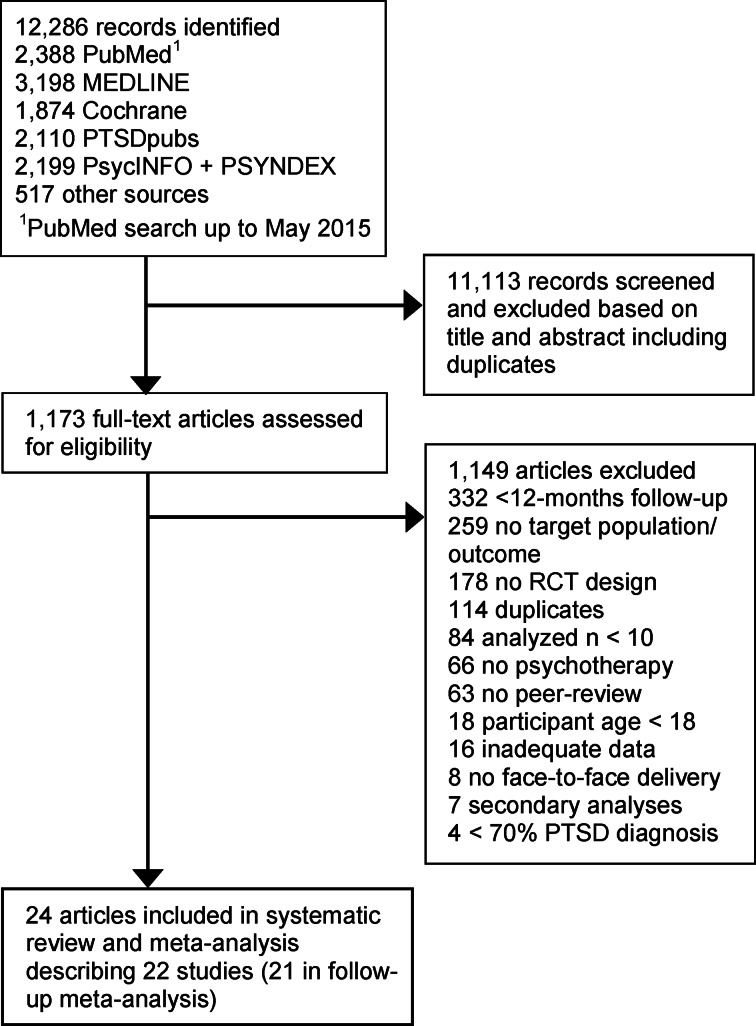
Long-term outcomes of psychological treatment for PTSD.

The search yielded 12 286 hits. Twenty-two eligible studies were included in this meta-analysis (Fig. 1). Studies were published between 1999 and 2018 and comprised 28–353 participants per study sample (*N* = 2638; *Median* = 101; Table 1). Participants (53% female) were on average 40 years old and met DSM-III/IV diagnostic criteria for PTSD (96%). Studies assessed civilian (64%) and military populations (32%) in individual-based treatment (86%). Nine studies (41%) quantified that 31–100% of participants received additional psychotropic medication during treatment. Five studies (23%) reported a stable medication dose for > 30 days at study entry for inclusion, and two studies (9%) excluded participants receiving any additional medication.

**Table 1.** Study characteristics of included randomized controlled trials with at least 12 months follow-up (LFU)

Study (year)	Population (♀, PTSD)	Trauma type	FU	Outcome measure	Treatment (n pre, post, FU)	Type	Format (n session)
Acierno et al. (2016)^a^	Military (6%, 77%)	Combat	12	PCL–M, BDI	BA + E (121, 79, 71), BA + E TH^b^	TF, –	Individual (8)
Blanchard et al. (2004)	Civilian (73%, 83%)	Accident	24	CAPS, BDI	CBT (37, 27, 22), SC (36, 27, 17) WL^b^	TF, control, –	Individual (10)
Cottraux et al. (2008)	Civilian (76%, 100%)	Mixed	20	PCL–C, BDI	CBT (30, 27, 16), SC (28, 15, 9)	TF, control	Individual (16)
Dunn et al. (2007	Military (0%, 100%)	Combat	12	CAPS, BDI–II	SMT (51, 34, 29), EG (50, 44, 37)	NTF, control	Group (14)
Ertl et al. (2011)^a^	Civilian (50%, 100%)	Abduction, war	12	CAPS, MINI	NET (16, 15, 14), AC (16, 13, 13), WL^b^	TF, control, –	Individual (8)
Foa et al. (1999)	Civilian (100%, 100%)	Mixed assault	12	PSS–I, BDI	PE (25, 23, 16), SIT + PE (30, 22, 16), SIT (26, 19, 14), WL^b^	TF, TF, NTF, –	Individual (9)
Foa et al. (2005)	Civilian (100%, 100%)	Mixed assault	12	PSS–I, BDI	PE (79, 52, 42), PE + CR (74, 44, 47), WL^b^	TF, TF, –	Individual (10)
Haller et al. (2016)^a^	Military (8%, 100%)	Mixed	12	PCL–M, HDRS	CPT–M (51, 45, 31), ICBTcom (50, 44, 25)	TF, NTF	Combined (12)
Hensel-Dittmann et al. (2011)^a^	Civilian (n.r., 100%)	Torture, war	12	CAPS, HAM–D	NET (15, 12, 8), SIT (13, 11, 7)	TF, NTF	Individual (10)
Hien et al. (2009)^a^	Civilian (100%, 80%)	Mixed	12	CAPS	SS (176, 108, 111), EG (177, 113, 104)	NTF, Control	Group (6)
Hien et al. (2015)	Civilian (81%, 77%)	Mixed	12	CAPS	SS + Se (32, 28, 21), SS + Pla (37, 29, 22)	NTF, NTF	Individual (6)
Langkaas et al. (2017)	Civilian (58%, 100%)	Mixed	12	PSS–I, BDI–II	IR (34, 31, 31), PE (33, 30, 27)	TF, TF	Individual (6)
Mueser et al. (2015)^a^	Civilian (69%, 100%)	Mixed	12	CAPS, BDI–II	CR (104, 86, 83), B–CBT (97, 75, 73)	TF, Control	Individual (16) (3)
Nacash et al. (2011)	Military (0%, 100%)	Combat	12	PSS–I BDI	PE (15, 13, 13), TAU (15, 13, 9)	TF, Control	Individual (11, n.r.)
Neuner et al. (2004)^a^	Civilian (60%, 100%)	Mixed	12	PDS-I	NET (17, 15, 14), SC (14, 13, 13), EG (12, 12, 11)	TF, Control, Control	Individual (4) (1)
Power et al. (2002)	Civilian (42%, 100%)	Mixed	15	IOE, HADS	EMDR (39, 27),^c^ E + CR (37, 21),^c^ WL^b^	TF, TF, –	Individual (4, 6)
Resick et al. (2002, 2012)^a^	Civilian (100%, 100%)	Sexual assault	74	CAPS, BDI	CPT (62, 41, n.r.), PE (62, 40, n.r.), WL^b^	TF, TF, –	Individual (13)
Resick et al. (2015)^a^	Military (7%, 100%)	Combat	12	PSS–I, BDI–II	CPT–C (56, 41, 28), PCT (52, 45, 28)	TF, Control	Group (12)
Rothbaum et al. (2014)^a^	Military (5%, 100%)	Combat	12	CAPS	VR + C (53, 28, 18), VR + A (50, 35, 22), VR + Pla (53, 34, 20)	TF, TF, TF	Individual (5)
Sloan et al. (2018)^a^	Military (0%, 100%)	Mixed	12	CAPS, BDI–II	CBT (98, 74, n.r.), PCT (100, 88, n.r.)	TF, Control	Group (14)
Tarrier et al. (1999, 2004)	Civilian (42%, 100%)	Mixed	60	CAPS, BDI	IE (35, 29, 29), CT (37, 33, 25)	TF, TF	Individual (12)
Thompson-Hollands et al. (2018)^a^	Mixed (48%, 100%)	Mixed	12	CAPS, BDI–II	WET (63, 60, 57), CPT (63, 52)^c^	TF, TF	Individual (5, 12)

A, alprazolam; AC, academic catch-up; B–CBT, brief cognitive behavioral therapy; BA + E, behavioral activation and exposure; BA + E TH, behavioral activation and exposure telehealth-based; BDI–II, beck depression inventory–II; BDI, Beck depression inventory; C, d-cycloserine; CAPS, clinician-administered PTSD scale; CBT, cognitive behavioral therapy; CPT–M, cognitive processing therapy modified; CPT, cognitive processing therapy; CR, cognitive restructuring; EG, educational group therapy; EMDR, eye movement desensitization and reprocessing; HADS, hospital anxiety and depression scale – depression subscale; HAM–D, Hamilton depression scale; HDRS, Hamilton depression rating scale; ICBTcom, integrated cognitive behavioral treatment for comorbidities; IOE, impact of events scale; IR, imagery rescripting therapy; MINI, mini international neuropsychiatric interview; n, sample size; n session, average number of sessions; NET, narrative exposure therapy; NTF, non-trauma-focused treatment; PCL-C, PTSD checklist – civilian version; PCL-M, PTSD checklist – military version; PCLS, post-traumatic checklist scale; PCT, present-centered therapy; PDSi, posttraumatic diagnostic scale – Interview-based; PE, prolonged exposure; Pla, placebo; PSS-I, PTSD symptom scale – Interview; PTSD, posttraumatic stress disorder; SC, supportive counseling; Se, sertraline; SIT, stress inoculation training; SMT, self-management therapy; SS, seeking safety; TAU, treatment as usual; TF, trauma-focused treatment; VR, virtual reality exposure therapy; WET, written exposure therapy; WL, wait-list.

a We thank primary study authors for providing additional data and/or data subsets.

b Treatment condition was excluded from all meta-analyses.

c Treatment condition was excluded from follow-up meta-analysis.

Of 46 included treatment conditions at posttest, 35 were active treatments including 28 TFT (*k* = 27 TF-CBT, *k* = 1 EMDR), and 7 non-TFT (all CBT). The 11 active, non-directive control conditions consisted of TAU (*k* = 5), social counseling (*k* = 3), present-centered therapy (*k* = 2), and educational groups (*k* = 1). One of six wait-list conditions was available at follow-up, and thus all passive control conditions were removed from analyses. Three active treatment conditions had inadequate follow-up data (*k* = 2 TF-CBT, *k* = 1 EMDR), and remained for qualitative and pre-post analyses, resulting in 43 CBT-based conditions for primary analyses. The average follow-up lasted 18 months and ranged from 12 to 74 months. Five studies (23%) had more than 12 months of follow-up. The dropout rate in active treatments (24%, *k* = 30) did not significantly differ from active control groups (18%, *k* = 7, *p* = 0.32). Dropouts in TFT (25%, *k* = 24) did also not differ significantly from non-TFT (21%, *k* = 6, *p* = 0.42).

### Long-term treatment effects on PTSD severity

#### Within-group effect sizes

Across all active treatments, the pooled within-group effect size was large for PTSD severity from pretest to follow-up, *g* = 1.36, 95% CI (1.14–1.57), *p* < 0.001 (Table 2; see Appendix A for all forest plots). Non-directive control conditions showed a medium effect size for the same period, *g* = 0.59, 95% CI (0.14–1.04), *p* < 0.05. Active treatments were more effective in reducing PTSD severity compared to non-directive control conditions from pretest to follow-up, respectively (*p* < 0.01). In addition, TFT and non-TFT types both yielded large within-group effect sizes from pretest to follow-up *g* = 1.44, 95% CI (1.20–1.67), *p* < 0.001; *g* = 1.08, 95% CI (0.52–1.63), *p* < 0.001, and effect sizes did not significantly differ from each other (*p* = 0.24). From posttest to follow-up, PTSD symptoms slightly improved in the active treatment and control conditions, *g* = 0.33 95% CI (0.23–0.44), *p* < 0.001; *g* = 0.16, 95% CI (0.04–0.28), *p* < 0.05 (see Appendix A). From pretest to posttest, within-effect sizes were large for active treatments and medium for control groups, *g* = 1.01, 95% CI (0.87–1.14), *p* < 0.001; *g* = 0.52, 95% CI (0.16–0.87), *p* < 0.01.

**Table 2.** Within-effect sizes (pretest – follow-up) and subgroup analyses for PTSD severity

	*k*	*g*	95% CI	*p*^a^	*Q*	*p*^b^	*I*^2^	*p*^c^
Condition								<0.01
Active treatment	32	1.36	1.14–1.57	<0.001	194.34	<0.001	84.05	
Active control	11	0.59	0.14–1.04	<0.05	132.38	<0.001	92.45	
Passive control	1	–						
Treatment type^d^								0.24
TFT	25	1.44	1.20–1.67	<0.001	132.08	<0.001	81.83	
Non-TFT	7	1.08	0.52–1.63	<0.001	62.21	<0.001	90.36	
Population								<0.01
Military	10	0.93	0.69–1.18	<0.001	32.38	<0.001	72.21	
Civilian	21	1.58	1.30–1.86	<0.001	102.89	<0.001	80.56	
Proportion female								
<50%	12	1.04	0.79–1.30	<0.001	47.23	<0.001	76.71	<0.001
⩾50%	18	1.62	1.34–1.90	<0.001	81.64	<0.001	79.18	
PTSD measure								<0.001
Interview-based	28	1.44	1.20–1.67	<0.001	158.82	<0.001	83.00	
Self-rated	4	0.80	0.53–1.07	<0.001	5.16	0.16	41.91	
Format								0.35
Group	4	1.00	0.18–1.82	<0.001	47.11	<0.001	93.63	
Individual	28	1.41	1.18–1.63	<0.001	146.60	<0.001	81.58	
Number of sessions								0.71
<10 sessions	14	1.31	1.05–1.57	<0.001	61.40	<0.001	78.82	
⩾10 sessions	18	1.39	1.04–1.75	<0.001	132.88	<0.001	87.21	
Type of analysis^1^								0.43
Intention-to-treat	18	1.42	1.15–1.70	<0.001	112.05	<0.001	84.83	
Completer	14	1.25	0.94–1.57	<0.001	59.30	<0.001	78.08	
Follow-up duration								0.05
12 months	26	1.26	1.03–1.49	<0.001	157.48	<0.001	84.13	
>12 months	6	1.77	1.33–2.21	<0.001	14.84	<0.05	66.32	

*Note.* Analyses based on 21 studies (20 for proportion female).

*p*-values less than 0.05 represent statistical significance. 95% CI *=* 95% confidence intervals, *g* = Hedges’ *g*, *k* = number of comparisons, PTSD *=* posttraumatic stress disorder.

1 Additional subgroup analyses for PTSD indicated significantly larger treatment effects for intention-to-treat samples with higher compared to lower dropout rates (⩾20%; <20%, *p* < 0.01), while treatment effects remained unaffected by losses to follow-up (<40%; ⩾40%, *p* = 0.61). In completer samples, attrition rates had no impact on treatment outcomes (*p* = 0.56; *p* = 0.34).

a *p*-value of Hedges’ *g*.

b *p*-value of *Q*-statistics.

c *p*-value between groups.

d Analyses include active treatments only.

Heterogeneity was large for within-group effect sizes from pretest to follow-up across condition and treatment types (*Q* > 62, *p* < 0.001, *I*^2^ > 81, Table 2). None of the moderators examined for PTSD severity – except for self-rated PTSD measure (*Q* = 5.16, *p* = 0.16, *I*^2^ = 41.91) – increased homogeneity in active treatments. However, subgroup analyses showed higher effect sizes for civilian compared to military populations (*p* < 0.01), for studies with larger proportions of female participants (*p* < 0.001), and for interview-based compared to self-rated outcome measures (*p* < 0.001). Subgroups did not significantly differ regarding treatment format, number of sessions, type of analysis used, or follow-up duration.

#### Between-group effect sizes

The pooled between-group effect size comparing active treatments to non-directive control groups was small for improved PTSD severity at follow-up, *g* = 0.42, 95% CI (0.15–0.68), *p* < 0.001 (Table 3). TFT showed a medium controlled effect size compared with control groups at follow-up, *g* = 0.51, 95% CI (0.15-0.86), *p* < 0.05. The effect size comparing TFT with non-TFT at follow-up was small and did not reach statistical significance, *g* = 0.35, 95% CI (−0.03 to 0.74), *p* = 0.07. At posttest, between-group effect sizes were small favoring active treatments and TFT over control groups, *g* = 0.24, 95% CI (0.04–0.44), *p* < 0.05; *g* = 0.25, 95% CI (0.03–0.47), *p* < 0.05 (see Appendix A).

**Table 3.** Between-effect sizes at follow-up

	*k*	*g*	95% CI	*p*^a^	*Q*	*p*^b^	*I*^2^
PTSD severity							
Treatment *v.* control	10	0.42	0.15–0.68	<0.001	29.76	<0.001	69.76
TFT *v.* control	8	0.51	0.15–0.86	<0.05	26.68	<0.001	73.76
TFT *v.* Non-TFT	3	0.35	−0.03 to 0.74	0.07	0.47	0.79	0.00
Non-TFT *v.* control	2	–					
Comorbid depressive symptoms							
Treatment *v.* control	7	0.15	−0.02 to 0.31	0.08	3.25	0.78	0.00
TFT *v.* control	6	0.14	−0.03 to 0.32	<0.05	85.27	<0.001	92.96
TFT *v.* Non-TFT	3	0.10	−0.38 to 0.58	0.68	2.73	0.25	26.83
Non-TFT *v.* control	1	–					

*Note.* 95% CI *=* 95% confidence intervals, *g* = Hedges’ *g*, *k* = number of comparisons, PTSD *=* posttraumatic stress disorder, TFT = trauma-focused treatment.

a *p*-value of Hedges’ *g*.

b *p*-value of *Q*-statistics.

Heterogeneity was moderate for between-condition effect sizes at follow-up (*Q* > 26, *p* < 0.001, *I*^2^ > 71), and low in the treatment type comparison (*Q* = 0.47, *p* = 0.79; *I*^2^ = 0, *k* = 3).

### Long-term treatment effects on comorbid depressive symptoms

#### Within-group effect sizes

Active treatments demonstrated a medium within-group effect size for reduced depressive symptoms from pretest to follow-up, *g* = 0.73, 95% CI (0.55–0.71), *p* < 0.001 (Table 4). Non-directive control conditions showed a small effect size from pretest to follow-up, *g* = 0.34, 95% CI (0.11–0.58), *p* < 0.01, which was significantly lower compared to the effect size of active treatments for the same period (*p* < 0.05). Within-group effect sizes were medium for TFTs, *g* = 0.78, 95% CI (0.58–0.99), *p* < 0.01, and small for non-TFTs from pretest to follow-up, *g* = 0.45, 95% CI (0.17–0.74), *p* < 0.01. However, this contrast was statistically not significant (*p* = 0.06). From posttest to follow-up, depressive symptoms remained stable in active treatments and control conditions, *g* = 0.10, 95% CI (−0.01 to 0.21), *p* = 0.08; *g* = 0.24, 95% CI (0.09–0.38), *p* < 0.001 (see Appendix A). Effect sizes from pretest to posttest were medium for active treatments and small for control groups, *g* = 0.68, 95% CI (0.55–0.80), *p* < 0.001; *g* = 0.24, 95% CI (0.09–0.39), *p* < 0.01.

**Table 4.** Within-effect sizes (pretest, follow-up) and subgroup analyses for comorbid depressive symptoms

	*k*	*g*	95% CI	*p*^a^	*Q*	*p*^b^	*I*^2^	*p*^c^
Condition								<0.05
Active treatment	24	0.73	0.55–0.71	<0.001	100.79	<0.001	77.18	
Active control	7	0.34	0.11–0.58	<0.01	32.36	<0.001	81.46	
Passive control	1	–						
Treatment type^d^								0.06
TFT	20	0.78	0.58–0.99	<0.01	93.25	<0.001	79.63	
Non-TFT	4	0.45	0.17–0.74	<0.01	4.54	0.21	33.91	

*Note.* 95% CI *=* 95% confidence intervals, *g* = Hedges’ *g*, *k* = number of comparisons, PTSD *=* posttraumatic stress disorder.

a *p*-value of Hedges’ *g*.

b *p*-value of *Q*-statistics.

c *p*-value between groups.

d Analyses include active treatments only.

All within-group effect sizes were largely heterogeneous at follow-up (*Q* > 33, *p* < 0.001; *I*^2^ > 76, see Table 3), except for non-TFTs (*Q* = 4.54, *p* = 0.21; *I*^2^ = 33.91, *k* = 4).

#### Between-group effect sizes

Table 3 reports small and non-significant between-group effect sizes of active treatments compared with active control conditions for comorbid depressive symptoms at follow-up, *g* = 0.15, 95% CI (−0.02 to 0.31), *p* = 0.08. Effect sizes of TFT were small compared with active control groups, *g* = 0.14, 95% CI (−0.03 to 0.32), *p* < 0.05, and did not differ statistically significant from non-TFT at follow-up, *g* = 0.10, 95% CI (−0.38 to 0.58), *p* = 0.68. At posttest, again between-group effect sizes for active treatments and TFTs were small compared with control groups, *g* = 0.30, 95% CI (0.06–0.53), *p* < 0.05; *g* = 0.32, 95% CI (0.04–0.59), *p* = 0.12 (see Appendix A).

Heterogeneity of between-condition effect sizes was low (*Q* *<* *4, p* > 0.05*; I*^2^ < 27) to large (*Q* > 30, *p* < 0.001; *I*^2^ > 80, see Table 3).

### Study quality

The overall risk of bias from studies included in this systematic review and meta-analysis ranged from low (50%) and unclear (24%) to high (26%) across all bias domains (see Appendix A for details per study). Most studies generated a low risk of bias concerning sequence generation (68%, *k* = 15), allocation concealment (50%, *k* = 11), and blinding of outcome assessors (90%, *k* = 20). Nine studies (41%) provided complete outcome data at follow-up indicating a low risk of bias. Nine studies (41%) also registered or published their study protocol a priori and adhered to it, while in half of the studies (50%) selective outcome reporting remained unclear. Few studies applied blinding of participants and personnel (9%, *k* = 2), and only one study had a low risk of bias in all domains.

### Additional analyses

The replaced correlations using *r* = 0.2 and *r* = 0.8 revealed marginally altered within-group effect sizes for active treatments and control conditions in all comparisons (see Appendix A for sensitivity analyses).

Publication bias remained untested due to moderate or large heterogeneity between effect sizes and small number of studies in our datasets (Ioannidis & Trikalinos, 2007; Rothstein et al., 2005; Sterne et al., 2006; Terrin et al., 2003).

## Discussion

### Summary of evidence

This systematic review and meta-analysis of 22 randomized controlled trials indicate that psychological treatment for adults with PTSD is efficacious in the long term. Active treatments yielded large symptom reductions of PTSD from pretest up to at least 12 months after initial treatment. Small treatment effects favored psychological treatment over non-directive control groups at follow-up, and symptom improvements remained stable from posttest to follow-up. TFT and non-TFT yielded large sustained improvements in PTSD from pretest to follow-up. Treatment effects of TFT were medium relative to non-directive control groups, and not significantly different from non-TFT at follow-up. Effect size estimates were of considerable heterogeneity and the number of available comparisons was low.

The large and stable within-group effect sizes of psychological treatment for PTSD in this meta-analysis are comparable with previous results at both short-term and medium-term follow-up (Ehring et al., 2014; Kline et al., 2018), yet uncontrolled comparisons must be interpreted cautiously. The between-group effect size of psychological treatment compared with active control groups is smaller than reported in a previous study with pooled wait-list and active control groups as comparator at follow-up (van Dis et al., 2020). However, the between-group effect size of TFT relative to active control groups is consistent with the previous finding. All active treatments included cognitive behavioral therapy (CBT) with TFT most frequently (78%) studied long-term, which is in line with previous evidence at medium-term follow-up (e.g. Kline et al., 2018). The enduring treatment effects of trauma-focused CBT after an average of 18 months confirm its recommendation as first-line therapy for PTSD (e.g. APA, 2017). One follow-up study was available on EMDR, yet the long-term data were insufficient to be included for meta-analysis. This finding highlights its current weaker empirical support as PTSD treatment (APA, 2017), and lasting benefits beyond CBT outcomes require future research. A few studies examined non-TFT showing large and enduring benefits for PTSD, which mirrors prior short-term findings regarding its empirical support and efficacy (e.g. Ehring et al., 2014; Lenz et al., 2017; Powers et al., 2010). Effect sizes of non-TFT were smaller compared with TFT at follow-up as previously reported (Ehring et al., 2014), but the difference was statistically non-significant. Given the small number of comparisons for non-TFT in particular, non-significant results should be interpreted as the absence of statistical evidence rather than as evidence of non-inferiority (Rief & Hofmann, 2018).

The within-group effect sizes of psychological treatments for improved comorbid depressive symptoms were medium from pretest to follow-up (or posttest), and smaller than previous findings at short-term follow-up on treatments aiming to reduce depression or PTSD (Morina, Malek, Nickerson, & Bryant, 2017). Between-group effect sizes at follow-up were also smaller than in the previous study mainly comparing active treatments to waitlist groups. Studies less frequently (73%) reported secondary depression outcomes at follow-up, and comparisons for depressive symptoms were likely underpowered.

The included studies differed widely across sample-related and treatment-related characteristics, and meta-analyses including the present one are inherently associated with heterogeneous effect sizes. In addition, dropout in active treatments was slightly higher compared to previous rates from mixed populations (Kline et al., 2018; Lewis, Roberts, Gibson, & Bisson, 2020), but lower than in military samples alone (Goetter et al., 2015). TFT and non-TFT types did not differ in dropout rates, which reflects some findings (e.g. Imel, Laska, Jakupcak, & Simpson, 2013; Thompson et al., 2018), and opposes others (Lewis et al., 2020).

Half of all studies (50%) were at high risk of bias in at least two domains potentially increasing the risk of overestimated treatment effects (e.g. Cuijpers, van Straten, Bohlmeijer, Hollon, & Andersson, 2010b). Subgroup analysis indicated large and non-significantly different effect sizes from ITT and completer samples at follow-up. However, in ITT samples (but not in completer samples) higher dropout was associated with larger effect sizes. This suggests that dropout is a critical source of bias that ITT analyses may not fully resolve assuming data are missing at random (White, Horton, Carpenter, & Pocock, 2011). In addition, treatment effects were significantly higher for studies using interview-based outcome measures compared to self-rated measures, challenging prior results from subgroup analyses in PTSD (Kline et al., 2018; Lambert & Alhassoon, 2015). As suggested for depression outcomes earlier, self-rated measures either underrated the improved symptoms, clinician-rated interviews were more sensitive to change, or a combination of both (Cuijpers, Li, Hofmann, & Andersson, 2010a).

The improved PTSD symptoms at follow-up in female and civilian populations compared with male and military subgroups replicate previous findings at short-term (Sloan et al., 2013; Watts et al., 2013) and medium-term follow-up (Kline et al., 2018; Wade et al., 2016). However, we noted that proportions of male participants were small in civilian populations but close to 100% in military samples, and future studies with balanced samples need to disentangle the effect of gender and population type. Importantly, treatment effects for PTSD did not differ regarding the length of follow-up duration, indicating that symptom improvements persisted beyond 12 months after treatment. The current number of studies with longer follow-up intervals is limited, and future evidence is required to confirm treatment gains beyond 12 months following treatment. Subgroup analyses on pre-specified moderators are exploratory and should be interpreted with caution as additional study-level factors may confound the results (Higgins & Green, 2011).

### Strengths and limitations

This systematic review and meta-analysis on long-term benefits of psychological treatment exceeds the number and variety of treatment studies included in former meta-analyses for PTSD (Kline et al., 2018; van Dis et al., 2020), and provides first evidence for comorbid depressive symptoms in PTSD at long-term. In addition, we performed a comprehensive literature search, adhered closely to the PRISMA recommendations, and examined pre-specified moderators of lasting treatment gains. However, there are several limitations to be noted. First, publication bias could not be tested due to small databases and considerable heterogeneity (Ioannidis & Trikalinos, 2007; Rothstein et al., 2005; Sterne et al., 2006; Terrin et al., 2003). Second, several studies had small sample sizes and included an unclear or high risk of bias. We analyzed two bias domains with no impact on effect sizes, but confidence in the results may remain limited. Third, the number of comparisons at follow-up was low, and specific treatment types beyond broad categories (i.e. TFT, non-TFT) remained untested. Finally, the studies provided few and inconsistent data on additional treatment (e.g. medication) during psychological treatment, and on whether participants received any treatment during the follow-up phase. It remains unclear if benefits can be ascribed to the initial psychological treatment alone.

## Conclusion

The results of this meta-analysis on psychological treatment for PTSD demonstrate large and sustained benefits after at least 12 months of follow-up. Active treatments were CBTs, and most studies examined TFT. Effect sizes of TFT were large for improved PTSD and medium for comorbid depressive symptoms from pretest to follow-up and superior to active control groups at follow-up. Non-TFT showed small to large benefits for PTSD and depressive symptoms from pretest to follow-up, respectively, but the number of available studies was scarce. Future well-designed studies are essential to determine the sustained gains from specific CBT types and other psychological treatments, and to confirm benefits beyond 12 months following initial treatment.

## Supplementary material

For supplementary material accompanying this paper visit https://doi.org/10.1017/S003329172100163X. click here to view supplementary material
