# Long-term Outcomes of Psychological Treatment for Posttraumatic Stress Disorder: A Systematic Review and Meta-Analysis - Corrigendum

**DOI:** 10.1017/S0033291721003214

**Published:** 2021-12

**Authors:** Maxi Weber, Sarah Schumacher, Wiebke Hannig, Jürgen Barth, Annett Lotzin, Ingo Schäfer, Thomas Ehring, Birgit Kleim

**Affiliations:** 1 Division of Clinical Psychology and Psychotherapy, Freie Universität Berlin, Berlin, Germany; 2 Division of Clinical Psychological Intervention, Freie Universität Berlin, Berlin, Germany; 3 Clinical Psychology and Psychotherapy, Health and Medical University, Potsdam, Germany; 4 Clinical Psychology and Psychotherapy, Department of Psychology, Philipps University of Marburg, Marburg, Germany; 5 Institute for Complementary and Integrative Medicine, University Hospital Zurich and University of Zurich, Zurich, Switzerland; 6 Department of Psychiatry and Psychotherapy, University Medical Center Hamburg-Eppendorf, Hamburg, Germany; 7 Department of Psychology, LMU Munich, Munich, Germany; 8 Department of Psychology, University of Zurich, Zurich, Switzerland; 9 Department of Psychiatry, Psychotherapy and Psychosomatics, Psychiatric University Hospital Zurich, University of Zurich, Zurich, Switzerland

This article was published in Psychological Medicine with errors in the author affiliations. This has now been corrected online and in the article.

The authors apologise for this error.
