# Supplementary material for: Long-term outcomes of psychological treatment for posttraumatic stress disorder: a systematic review and meta-analysis
Source: Psychol Med. 2021 Jun 28;51(9):1420–30. doi: 10.1017/S003329172100163X (PMC8311818; doi:10.1017/S003329172100163X)
Supplement: Supplementary file 1 [file S003329172100163Xsup001.docx]

**Appendix A. Supplementary data**

Bibliography of studies included in this meta-analysis

Acierno, R., Gros, D. F., Ruggiero, K. J., Hernandez-Tejada, B. M. A., Knapp, R. G., Lejuez, C. W., … Tuerk, P. W. (2016). Behavioral activation and therapeutic exposure for posttraumatic stress disorder: A noninferiority trial of treatment dilvered in person versus home-based tele heath. *Depression and Anxiety*, *33*(5), 415–423. doi:10.1002/da.22476

Blanchard, E. B., Hickling, E. J., Malta, L. S., Freidenberg, B. M., Canna, M. A., Kuhn, E., …Galovski, T. E. (2004). One- and two-year prospective follow-up of cognitive behavior therapy or supportive psychotherapy. *Behaviour Research and Therapy*, *42*(7), 745–759. doi:10.1016/S0005-7967(03)00201-8

Cottraux, J., Note, I., Yao, S. N., De Mey-Guillard, C., Bonasse, F., Djamoussian, D., … Chen, Y. (2008). Randomized controlled comparison of cognitive behavior therapy with rogerian supportive therapy in chronic post-traumatic stress disorder: A 2-year follow-up. *Psychotherapy and Psychosomatics*, *77*(2), 101–110. doi:10.1159/000112887

Dunn, N. J., Rehm, L. P., Schillaci, J., Souchek, J., Mehta, P., Ashton, C. M., … Hamilton, J. D. (2007). A randomized trial of self-management and psychoeducational group therapies for comorbid chronic posttraumatic stress disorder and depressive disorder. *Journal of Traumatic Stress*, *20*(3), 221–237. doi:10.1002/jts.20214

Ertl, V., Pfeiffer, A., Schauer, E., Elbert, T., & Neuner, F. (2011). Community-Implemented Trauma Therapy for Former Child Soldiers in Northern Uganda. *JAMA*, *306*(5), 503–512. doi:10.1001/jama.2011.1060

Foa, E. B., Dancu, C. V, Hembree, E. A., Jaycox, L. H., Meadows, E. A., & Street, G. P. (1999). A comparison of exposure therapy, stress inoculation training, and their combination for reducing posttraumatic stress disorder in female assault victims. *Journal of Consulting and Clinical Psychology*, *67*(2), 194–200. doi: 10.1037//0022-006x.67.2.194

Foa, E. B., Hembree, E. A., Cahill, S. P., Rauch, S. A. M., Riggs, D. S., Feeny, N. C., & Yadin, E. (2005). Randomized trial of prolonged exposure for posttraumatic stress disorder with and without cognitive restructuring: Outcome at academic and community clinics. *Journal of Consulting and Clinical Psychology*, *73*(5), 953–964. doi:10.1037/0022-006X.73.5.953

Haller, M., Norman, S. B., Cummins, K., Trim, R. S., Xu, X., Cui, R., … Tate, S. R. (2016). Integrated Cognitive Behavioral Therapy Versus Cognitive Processing Therapy for Adults With Depression, Substance Use Disorder, and Trauma. *Journal of Substance Abuse Treatment*, *62*, 38–48. doi:10.1016/j.jsat.2015.11.005

Hensel-Dittmann, D., Schauer, M., Ruf, M., Catani, C., Odenwald, M., Elbert, T., & Neuner, F. (2011). Treatment of traumatized victims of war and torture: A randomized controlled comparison of narrative exposure therapy and stress inoculation training. *Psychotherapy and Psychosomatics*, *80*(6), 345–352. doi:10.1159/000327253

Hien, D. A., Levin, F. R., Ruglass, L. M., López-Castro, T., Papini, S., Hu, M.-C., … Herron, A. (2015). Combining seeking safety with sertraline for PTSD and alcohol use disorders: A randomized controlled trial. *Journal of Consulting and Clinical Psychology*, *83*(2), 359–369. doi:10.1037/a0038719

Hien, D. A., Wells, E. A., Jiang, H., Suarez-Morales, L., Campbell, A. N. C., Cohen, L. R., … Nunes, E. V. (2009). Multisite randomized trial of behavioral interventions for women with co-occurring PTSD and substance use disorders. *Journal of Consulting and Clinical Psychology*, *77*(4), 607–619. doi:10.1037/a0016227

Langkaas, T. F., Hoffart, A., Øktedalen, T., Ulvenes, P. G., Hembree, E. A., & Smucker, M. (2017). Exposure and non-fear emotions: A randomized controlled study of exposure-based and rescripting-based imagery in PTSD treatment. *Behaviour Research and Therapy*, *97*, 33–42. doi:10.1016/j.brat.2017.06.007

Mueser, K. T., Rosenberg, S. D., Wolfe, R. S., Xie, H., Silverstein, S. M., Lu, W., … Duva, S. M. (2015). Evaluation of cognitive restructuring for post-traumatic stress disorder in people with severe mental illness. *British Journal of Psychiatry*, *206*(06), 501–508. doi:10.1192/bjp.bp.114.147926

Nacasch, N., Foa, E. B., Huppert, J. D., Tzur, D., Fostick, L., Dinstein, Y., … Zohar, J. (2011). Prolonged exposure therapy for combat- and terror-related posttraumatic stress disorder: A randomized control comparison with treatment as usual. *Journal of Clinical Psychiatry*, *72*(9), 1174–1180. doi:10.4088/JCP.09m05682blu

Neuner, F., Schauer, M., Klaschik, C., Karunakara, U., & Elbert, T. (2004). A comparison of narrative exposure therapy, supportive counseling, and psychoeducation for treating posttraumatic stress disorder in an African refugee settlement. *Journal of Consulting and Clinical Psychology*, *72*(4), 579–587. doi:10.1037/0022-006X.72.4.579

Power, K., McGoldrick, T., Brown, K., Buchanan, R., Sharp, D., Swanson, V., & Karatzias, A. (2002). A controlled comparison of eye movement desensitization and reprocessing versus exposure plus cognitive restructuring versus waiting list in the treatment of post-traumatic stress disorder. *Clinical Psychology & Psychotherapy*, *9*(5), 299–318. doi:10.1002/cpp.341

Resick, P. A., Nishith, P., Weaver, T. L., Astin, M. C., & Feuer, C. A. (2002). A comparison of cognitive-processing therapy with prolonged exposure and a waiting condition for the treatment of chronic posttraumatic stress disorder in female rape victims. *Journal of Consulting and Clinical Psychology*, *70*(4), 867–879. doi:10.1037/0022-006X.70.4.867

Resick, P. A., Williams, L. F., Suvak, M. K., Monson, C. M., & Gradus, J. L. (2012). Long-term outcomes of cognitive–behavioral treatments for posttraumatic stress disorder among female rape survivors. *Journal of Consulting and Clinical Psychology*, *80*(2), 201–210. doi:10.1037/a0026602

Rothbaum, B. O., Price, M., Jovanovic, T., Norrholm, S. D., Gerardi, M., Dunlop, B., … Ressler, K. J. (2014). A Randomized, Double-Blind Evaluation of d -Cycloserine or Alprazolam Combined With Virtual Reality Exposure Therapy for Posttraumatic Stress Disorder in Iraq and Afghanistan War Veterans. *American Journal of Psychiatry*, *171*(6), 640–648. doi:10.1176/appi.ajp.2014.13121625

Sloan, D., Marx, B. P., Lee, D.J., & Resick, P. A. (2018). A Brief Exposure-Based Treatment vs Cognitive Processing Therapy for Posttraumatic Stress Disorder: A Randomized Noninferiority Clinical Trial. *JAMA Psychiatry 75*(3), 233–239. doi:10.1001/jamapsychiatry.2017.4249.

Sloan, D., Unger, W., Lee, D., & Beck, J. G. (2018). A Randomized Controlled Trial of Group Cognitive Behavioral Treatment for Veterans Diagnosed With Chronic Posttraumatic Stress Disorder: Group Cognitive Behavioral Treatment for PTSD. *Journal of Traumatic Stress. 31*(6), 886–898. doi:10.1002/jts.22338.

Tarrier, N., Sommerfeld, C., Pilgrim, H., & Humphreys, L. (1999). Cognitive therapy or imaginal exposure in the treatment of post-traumatic stress disorder. *British Journal of Psychiatry*, *175*(6), 571–575. doi:10.1192/bjp.175.6.571

Tarrier, N., & Sommerfield, C. (2004). Treatment of chronic PTSD by cognitive therapy and exposure: 5-year follow-up. *Behavior Therapy*, *35*(2), 231–246. doi:10.1016/S0005-7894(04)80037-6

Thompson-Hollands, J., Marx, B., Lee, D., Resick, P., & Sloan, D. (2018). Long‐term treatment gains of a brief exposure‐based treatment for PTSD. *Depression and Anxiety, 35*(10), 985–991. doi:10.1002/da.22825

| Table A1  *PRISMA Checklist* | | | |
| --- | --- | --- | --- |
| Section/topic | Item No | Checklist item | Reported at paragraph |
| *Title* |  |  |  |
| Title | 1 | Identify the report as a systematic review, meta-analysis, or both | Title page |
| *Abstract* |  |  |  |
| Structured summary | 2 | Provide a structured summary including, as applicable, background, objectives, data sources, study eligibility criteria, participants, interventions, study appraisal and synthesis methods, results, limitations, conclusions and implications of key findings, (systematic review registration number) | Title page |
| *Introduction* | |  |  |
| Rationale | 3 | Describe the rationale for the review in the context of what is already known | Introduction:  1–4 |
| Objectives | 4 | Provide an explicit statement of questions being addressed with reference to participants, interventions, comparisons, outcomes, and study design (PICOS) | Introduction:  5 |
| *Methods* |  |  |  |
| Protocol and registration | 5 | Indicate if a review protocol exists, if and where it can be accessed (such as web address), and, if available, provide registration information including registration number | Method:  1 |
| Eligibility criteria | 6 | Specify study characteristics (such as PICOS, length of follow-up) and report characteristics (such as years considered, language, publication status) used as criteria for eligibility, giving rationale | Method:  2 |
| Information sources | 7 | Describe all information sources (such as databases with dates of coverage, contact with study authors to identify additional studies) in the search and date last searched | Method:  3 |
| Search | 8 | Present full electronic search strategy for at least one database, including any limits used, such that it could be repeated | Method:  3 |
| Study selection | 9 | State the process for selecting studies (that is, screening, eligibility, included in systematic review, and, if applicable, included in the meta-analysis) | Method:  3 |
| Data collection process | 10 | Describe method of data extraction from reports (such as piloted forms, independently, in duplicate) and any processes for obtaining and confirming data from investigators | Method:  4 |
| Data items | 11 | List and define all variables for which data were sought (such as PICOS, funding sources) and any assumptions and simplifications made | Method:  4–5, Appendix A |
| Risk of bias in individual studies | 12 | Describe methods used for assessing risk of bias of individual studies (including specification of whether this was done at the study or outcome level), and how this information is to be used in any data synthesis | Method:  6 |

| Table A1  *(continued)* | | | |
| --- | --- | --- | --- |
| Section/topic | Item No | Checklist item | Reported at paragraph |
| Summary measures | 13 | State the principal summary measures (such as risk ratio, difference in means). | Method:  7 |
| Synthesis of results | 14 | Describe the methods of handling data and combining results of studies, if done, including measures of consistency (such as I^2^ statistic) for each meta-analysis | Method:  7–8 |
| Risk of bias across studies | 15 | Specify any assessment of risk of bias that may affect the cumulative evidence (such as publication bias, selective reporting within studies) | Method:  10 |
| Additional analyses | 16 | Describe methods of additional analyses (such as sensitivity or subgroup analyses, meta-regression), if done, indicating which were pre-specified | Method:  8–9 |
| *Results* |  |  |  |
| Study selection | 17 | Give numbers of studies screened, assessed for eligibility, and included in the review, with reasons for exclusions at each stage, ideally with a flow diagram | Results:  1-2, Figure 1 |
| Study characteristics | 18 | For each study, present characteristics for which data were extracted (such as study size, PICOS, follow-up period) and provide the citations | Results:  1-2, Table 1, Appendix A |
| Risk of bias within studies | 19 | Present data on risk of bias of each study and, if available, any outcome-level assessment (see item 12). | Results:  11, Appendix A |
| Results of individual studies | 20 | For all outcomes considered (benefits or harms), present for each study (a) simple summary data for each intervention group and (b) effect estimates and confidence intervals, ideally with a forest plot | Results:  Table 2–4, Appendix A |
| Synthesis of results | 21 | Present results of each meta-analysis done, including confidence intervals and measures of consistency | Results:  3-10, Table 2–4 |
| Risk of bias across studies | 22 | Present results of any assessment of risk of bias across studies (see item 15) | Results:  11, 13 |
| Additional analysis | 23 | Give results of additional analyses, if done (such as sensitivity or subgroup analyses, meta-regression) (see item 16) | Results:  3-10, 12, Appendix A |
| *Discussion* |  |  |  |
| Summary of evidence | 24 | Summarise the main findings including the strength of evidence for each main outcome; consider their relevance to key groups (such as health care providers, users, and policy makers) | Discussion:  1-6 |
|  |  |  |  |

| Table A1  *(continued)* | | | |
| --- | --- | --- | --- |
| Section/topic | Item No | Checklist item | Reported at paragraph |
| Limitations | 25 | Discuss limitations at study and outcome level (such as risk of bias), and at review level (such as incomplete retrieval of identified research, reporting bias) | Discussion:  7 |
| Conclusions | 26 | Provide a general interpretation of the results in the context of other evidence, and implications for future research | Discussion:  8 |
| Funding |  |  |  |
| Funding | 27 | Describe sources of funding for the systematic review and other support (such as supply of data) and role of funders for the systematic review | First page:  1 |

| Table A2  *Study characteristics extracted from primary studies* | |
| --- | --- |
| *General information* | Study authors, year of publication, location of study implementation |
| *Sample-related characteristics* | Total sample size, sample sizes per treatment arm, age, sex, number/proportion of participants with clinical PTSD, trauma type, number/proportion of participants with comorbid depressive symptoms |
| *Treatment-related* characteristics | Treatment condition, treatment/control type, treatment format (e.g., group-based), setting (e.g. outpatient), largest follow-up interval, dropout rate or numbers of participants initiating and not completing the whole course of treatment, data representing PTSD and comorbid depressive symptoms |

| Table A3  *Within-group effect sizes and between-group effect sizes at posttest for PTSD severity* | | | | | | | |
| --- | --- | --- | --- | --- | --- | --- | --- |
|  | *k* | *g* | 95 % CI | *p*^‡^ | *Q* | *p*^§^ | *I*^2^ |
| *Within-group effect sizes* *(posttest – follow-up)* | | | |  |  |  |  |
| Condition |  |  |  |  |  |  |  |
| Psychotherapy | 32 | 0.33 | 0.23 – 0.44 | < .001 | 86.93 | < .001 | 64.34 |
| Active control | 11 | 0.16 | 0.04 – 0.28 | < .05 | 14.54 | .15 | 31.23 |
| *Within-group effect sizes (pretest – posttest)* | | | |  |  |  |  |
| Condition |  |  |  |  |  |  |  |
| Psychotherapy | 35 | 1.01 | 0.87 – 1.14 | < .001 | 145.00 | < .001 | 76.55 |
| Active control | 11 | 0.52 | 0.16 – 0.87 | < .01 | 96.64 | < .001 | 89.65 |
| Treatment Type^†^ |  |  |  |  |  |  |  |
| TFT | 28 | 1.04 | 0.91 – 1.17 | < .001 | 87.29 | < .001 | 69.07 |
| Non-TFT | 7 | 0.85 | 0.39 – 1.31 | < .001 | 57.71 | < .001 | 89.60 |
| *Between-group effect sizes (posttest)* | | |  |  |  |  |  |
| Treatment vs control | 10 | 0.24 | 0.04 – 0.44 | < .05 | 19.30 | < .05 | 53.36 |
| TFT vs control | 8 | 0.25 | 0.03 – 0.47 | < .05 | 19.29 | < .05 | 58.52 |
| TFT vs Non-TFT | 3 | 0.06 | -0.26 – 0.37 | .72 | 0.61 | 0.74 | 0.00 |
| Non-TFT vs control | 2 | – |  |  |  |  |  |
| *Note.* 95 % CI *=* 95% confidence intervals, *g* = Hedges’ *g*, *k* = number of comparisons, PTSD *=* posttraumatic stress disorder.  ^†^ Analyses include active treatments only.  ^‡^ P-value of Hedges’ *g*.  ^§^ P-value of *Q*-statistics. | | | | | | | |

| Table A4  *Within-group effect sizes and between-group effect sizes at posttest for comorbid depressive symptoms* | | | | | | | |
| --- | --- | --- | --- | --- | --- | --- | --- |
|  | *k* | *g* | 95 % CI | *p*^‡^ | *Q* | *p*^§^ | *I*^2^ |
| *Within-group effect sizes (posttest – follow-up)* | | | |  |  |  |  |
| Condition |  |  |  |  |  |  |  |
| Psychotherapy | 24 | 0.10 | -0.01– 0.21 | .08 | 56.02 | .00 | 58.94 |
| Active control | 7 | 0.24 | 0.09 – 0.38 | < .001 | 8.73 | .19 | 31.29 |
| *Within-group effect sizes (pretest – posttest)* | | | |  |  |  |  |
| Condition |  |  |  |  |  |  |  |
| Psychotherapy | 28 | 0.68 | 0.55 – 0.80 | < .001 | 93.18 | < .001 | 71.02 |
| Active control | 8 | 0.24 | 0.09 – 0.39 | < .01 | 16.11 | < .05 | 56.56 |
| Treatment Type^†^ |  |  |  |  |  |  |  |
| TFT | 24 | 0.71 | 0.57 – 0.84 | < .001 | 77.58 | < .001 | 70.36 |
| Non-TFT | 4 | 0.45 | 0.01 – 0.89 | < .05 | 12.34 | < .01 | 75.69 |
| *Between-group effect sizes* | |  |  |  |  |  |  |
| Treatment vs control | 8 | 0.30 | 0.06 – 0.53 | < .05 | 13.82 | .05 | 49.36 |
| TFT vs control | 7 | 0.32 | 0.04 – 0.59 | < .05 | 13.81 | < .05 | 56.55 |
| TFT vs Non-TFT | 3 | 0.28 | -0.17 – 0.73 | 0.23 | 3.64 | .16 | 45.05 |
| Non-TFT vs control | 1 | – |  |  |  |  |  |
| *Note.* 95 % CI *=* 95% confidence intervals, *g* = Hedges’ *g*, *k* = number of comparisons.  ^†^ Analyses include active treatments only.  ^‡^ P-value of Hedges’ *g*.  ^§^ P-value of *Q*-statistics. | | | | | | | |

| Table A5  *Risk of bias assessments for included studies* | | | | | | |
| --- | --- | --- | --- | --- | --- | --- |
| Study | Random sequence generation | Allocation  concealment | Blinding participants/ personnel | Blinding outcome assessors^†^ | Incomplete follow-up data | Selective outcome reporting |
| Acierno et al. (2016) | low | unclear | high | low | high | high |
| Blanchard et al. (2004) | unclear | unclear | high | low | high | unclear |
| Cottraux et al. (2008) | low | low | high | low | high | unclear |
| Dunn et al. (2007) | low | low | high | low | high | unclear |
| Ertl et al. (2011)^‡^ | unclear | unclear | high | low | high | low |
| Foa et al. (1999) | unclear | unclear | high | low | high | unclear |
| Foa et al. (2005) | low | unclear | high | low | high | unclear |
| Haller et al. (2016)^‡^ | unclear | unclear | high | low | high | low |
| Hensel-Dittmann et al. (2011) | low | low | high | unclear | low | unclear |
| Hien et al. (2009) | low | low | high | low | low | high |
| Hien et al. (2015) | low | low | low | low | high | low |
| Langkaas et al. (2017) | low | low | high | low | low | low |
| Mueser et al. (2015) | low | low | high | low | unclear | low |
| Nacash et al. (2011) | low | low | high | low | low | unclear |
| Neuner et al. (2004) | low | unclear | high | low | low | unclear |
| Power et al. (2002) | unclear | unclear | high | high^1^ | high | unclear |
| Resick et al. (2012) | unclear | unclear | high | low | low | unclear |
| Resick et al. (2015) | unclear | unclear | high | low | low | low |
| Rothbaum et al. (2014) | low | low | low | low | low | low |
| Sloan et al. (2018) | low | low | high | low | low | low |
| Tarrier et al. (2004) | low | low | high | low | high | unclear |
| Thompson et al. (2018) | low | unclear | high | low | unclear | low |
| *Note.* high = high risk of bias; low = low risk of bias; unclear = unclear/unknown risk of bias. ^1^Only pre-post data from the study could be included in this meta-analysis having a low risk of bias due to blinded interviewers. Follow-up assessments, however, were conducted by therapists unblinded to treatment allocation.  ^†^ The use of valid and reliable self-reported PTSD measures was rated as low risk of bias.  ^‡^ Study authors kindly provided data for subsamples. | | | | | | |

| Table A6  *Sensitivity analyses for missing within correlation coefficients of effect sizes* | | | | | | | |
| --- | --- | --- | --- | --- | --- | --- | --- |
|  | *k* | *g* | 95 % CI | *p*^†^ | *Q* | *p*^‡^ | *I*^2^ |
| *Pretest - follow-up* | | |  |  |  |  |  |
| *PTSD severity* |  |  |  |  |  |  |  |
| *r* = .2 |  |  |  |  |  |  |  |
| Active treatment | 32 | 1.36 | 1.15 – 1.56 | < .001 | 146.78 | < .001 | 78.88 |
| Active control | 11 | 0.63 | 0.20 – 1.05 | < .01 | 87.53 | < .001 | 88.58 |
| *r* = .8 |  |  |  |  |  |  |  |
| Active treatment | 32 | 1.34 | 1.12 – 1.57 | < .001 | 349.14 | < .001 | 91.12 |
| Active control | 11 | 0.57 | 0.11 – 1.04 | < .05 | 318.81 | < .001 | 96.86 |
| *Depressive symptoms* |  |  |  |  |  |  |  |
| *r* = .2 |  |  |  |  |  |  |  |
| Active treatment | 24 | 0.73 | 0.56 – 0.91 | < .001 | 78.68 | < .001 | 70.77 |
| Active control | 7 | 0.28 | 0.08 – 0.49 | < .01 | 22.41 | < .01 | 73.23 |
| *r* = .8 |  |  |  |  |  |  |  |
| Active treatment | 24 | 0.74 | 0.55 – 0.93 | < .001 | 166.27 | < .001 | 86.17 |
| Active control | 7 | 0.38 | 0.14 – 0.62 | < .01 | 60.31 | < .001 | 90.05 |
| *Posttest – follow-up* | |  |  |  |  |  |  |
| *PTSD severity* |  |  |  |  |  |  |  |
| *r* = .2 |  |  |  |  |  |  |  |
| Active treatment | 32 | 0.34 | 0.23 – 0.44 | < .001 | 55.79 | < .001 | 44.43 |
| Active control | 11 | 0.18 | 0.07 – 0.30 | < .001 | 10.74 | 0.38 | 6.86 |
| *r* = .8 |  |  |  |  |  |  |  |
| Active treatment | 32 | 0.32 | 0.23 – 0.42 | < .001 | 198.66 | < .001 | 84.40 |
| Active control | 11 | 0.12 | 0.02 – 0.23 | < .001 | 27.70 | < .001 | 63.90 |
| *Depressive symptoms* |  |  |  |  |  |  |  |
| *r* = .2 |  |  |  |  |  |  |  |
| Active treatment | 24 | 0.10 | -0.01 – 0.21 | .09 | 36.26 | < .05 | 36.57 |
| Active control | 7 | 0.24 | 0.10 – 0.38 | < .001 | 5.97 | 0.43 | 0.00 |
| *r* = .8 |  |  |  |  |  |  |  |
| Active treatment | 24 | 0.10 | -0.00 – 0.21 | .06 | 127.53 | < .001 | 81.97 |
| Active control | 7 | 0.24 | 0.10 – 0.38 | < .001 | 18.62 | < .001 | 67.78 |

| Table A6  *(continued)* | | | | | | | |
| --- | --- | --- | --- | --- | --- | --- | --- |
| *Pretest - posttest* | |  |  |  |  |  |  |
| *PTSD severity* |  |  |  |  |  |  |  |
| *r* = .2 |  |  |  |  |  |  |  |
| Active treatment | 35 | 1.00 | 0.87 – 1.13 | < .001 | 110.97 | < .001 | 68.27 |
| Active control | 11 | 0.53 | 0.19 – 0.87 | *<* .001 | 62.43 | < .001 | 83.98 |
| *r* = .8 |  |  |  |  |  |  |  |
| Active treatment | 35 | 0.98 | 0.84 – 1.11 | < .001 | 271.93 | < .001 | 87.50 |
| Active control | 11 | 0.48 | 0.12 – 0.85 | < .01 | 228.62 | < .001 | 95.63 |
| *Depressive symptoms* |  |  |  |  |  |  |  |
| *r* = .2 |  |  |  |  |  |  |  |
| Active treatment | 28 | 0.66 | 0.53 – 0.79 | < .001 | 74.75 | < .001 | 63.88 |
| Active control | 8 | 0.21 | 0.06 – 0.36 | < .01 | 12.79 | .01 | 45.28 |
| *r* = .8 |  |  |  |  |  |  |  |
| Condition |  |  |  |  |  |  |  |
| Active treatment | 28 | 0.67 | 0.55 – 0.80 | < .001 | 153.32 | < .001 | 82.39 |
| Active control | 8 | 0.27 | 0.13 – 0.42 | < .001 | 26.12 | < .01 | 73.20 |
| *Note.* 95 % CI *= ﻿*95% confidence intervals, *g* = Hedges’ *g*, *k* = number of comparisons, PTSD *=* posttraumatic stress disorder.  ^†^ P-value of Hedges’ *g*.  ^‡^ P-value of *Q*-statistics. | | | | | | | |

*
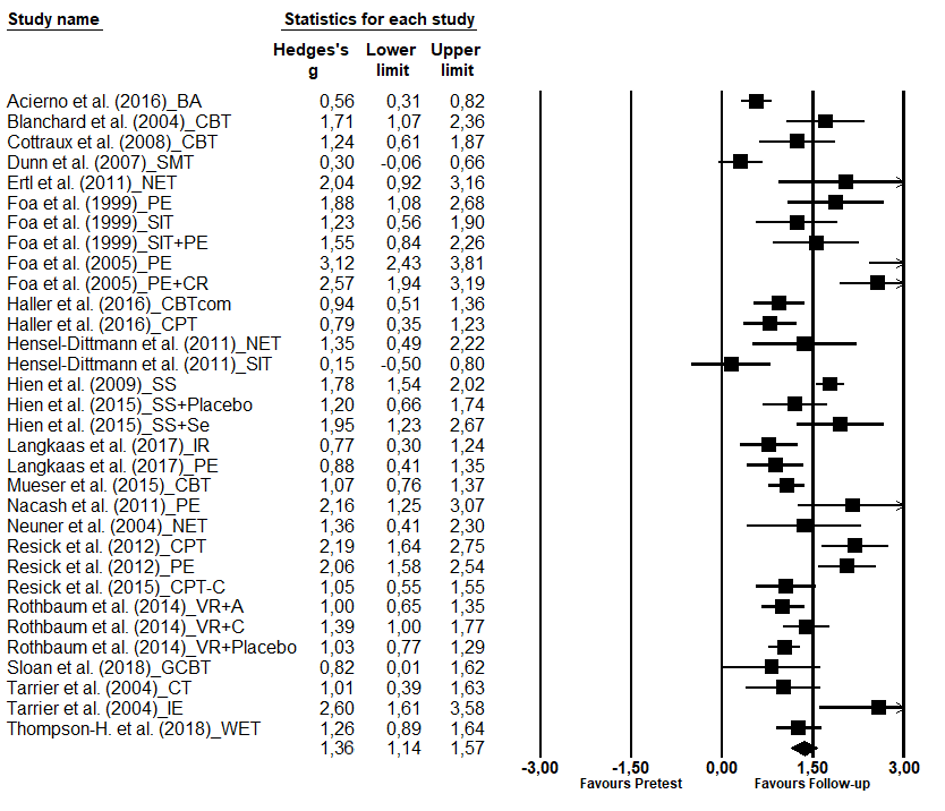
*


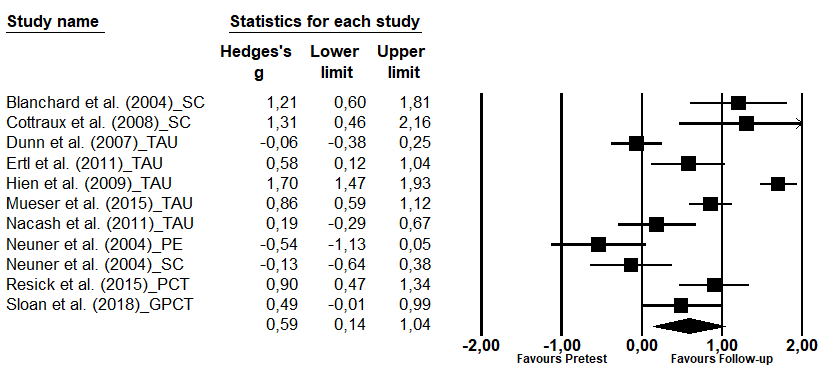


*Figure A1.* Within-group effect sizes (pretest – follow-up) of active treatments (top) and non-directive control groups (bottom) for PTSD severity.


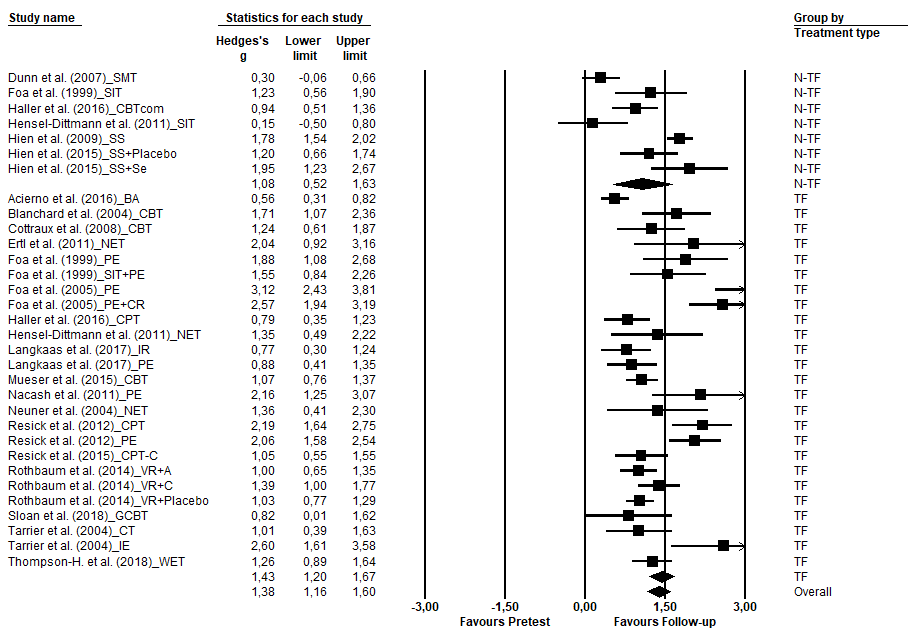


*Figure A2.* Within-group effect sizes (pretest – follow-up) of trauma-focused treatments (TFTs) and non-TFTs for PTSD severity.


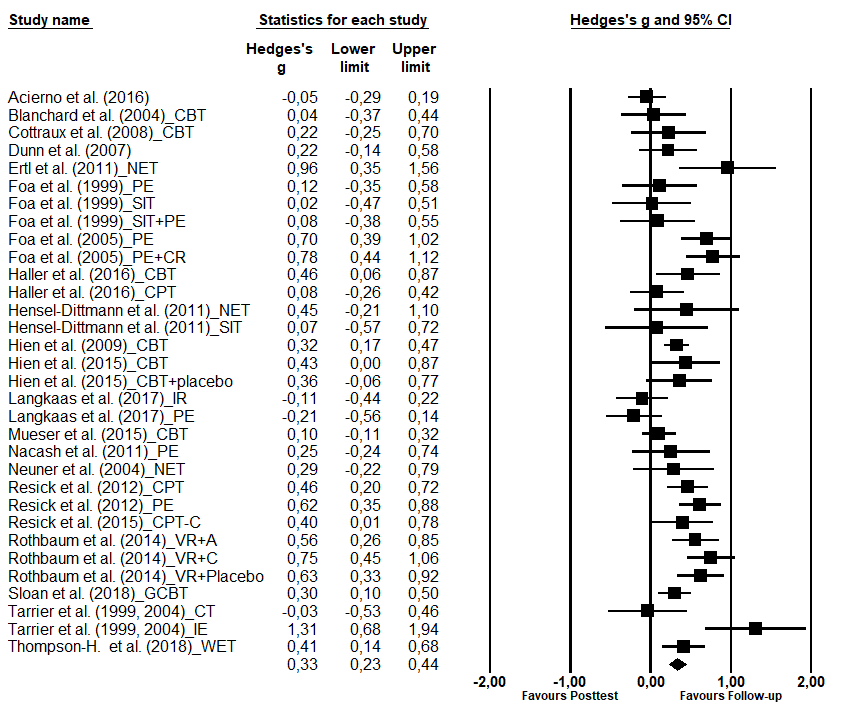


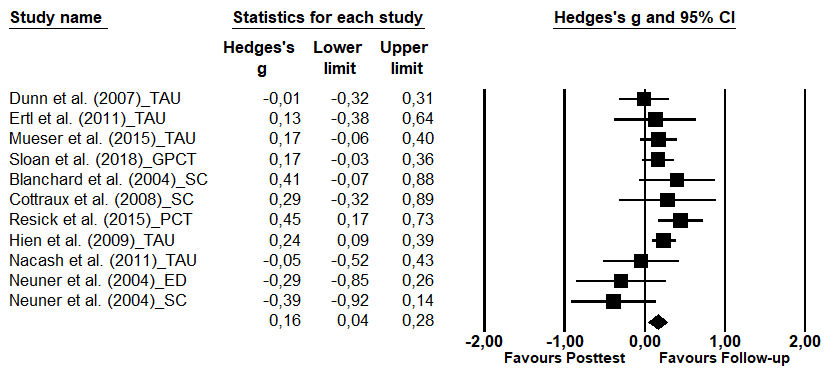


*Figure A3.* Within-group effect sizes (posttest – follow-up) of active treatments (top) and control conditions (bottom) for PTSD severity.

*
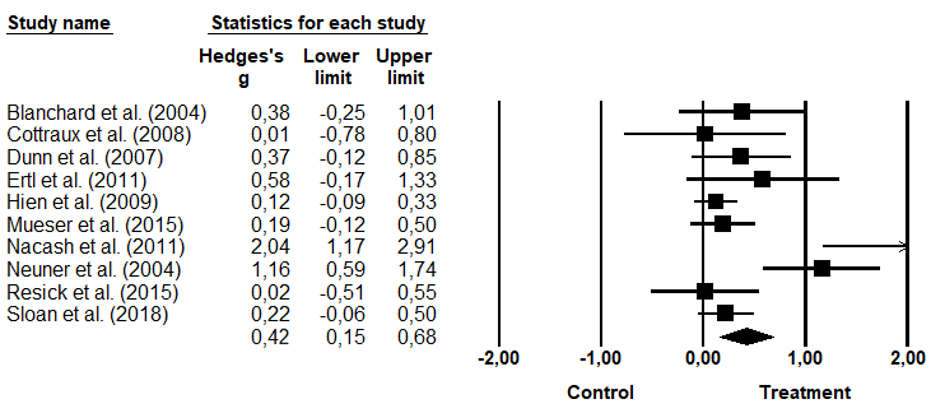
*

*
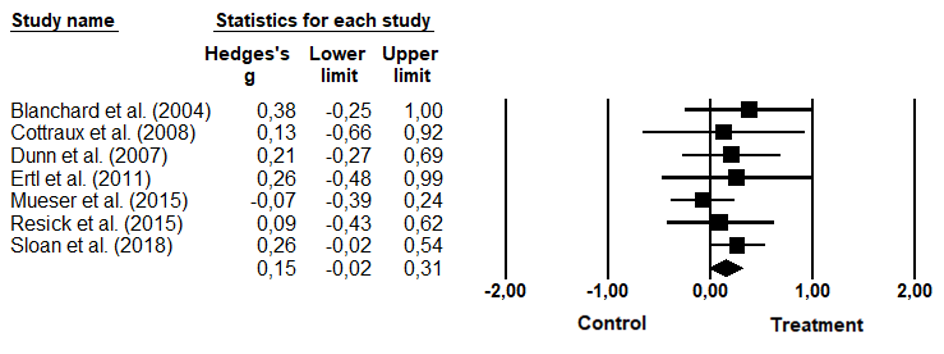
*

*Figure A4.* Between-group effect sizes for PTSD severity (top) and comorbid depressive symptoms (bottom) at follow-up comparing active treatments with non-directive control groups.


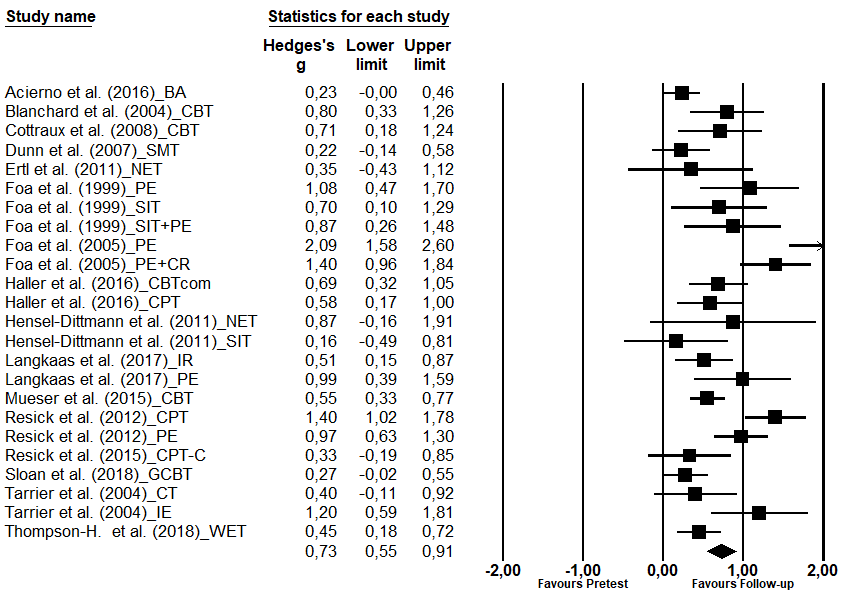


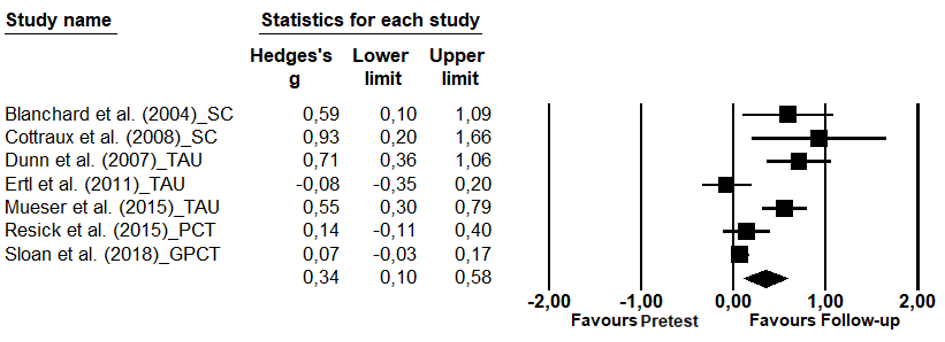


*Figure A5.* Within-group effect sizes (pretest – follow-up) of active treatments (top) and non-directive control groups (bottom) for comorbid depressive symptoms.


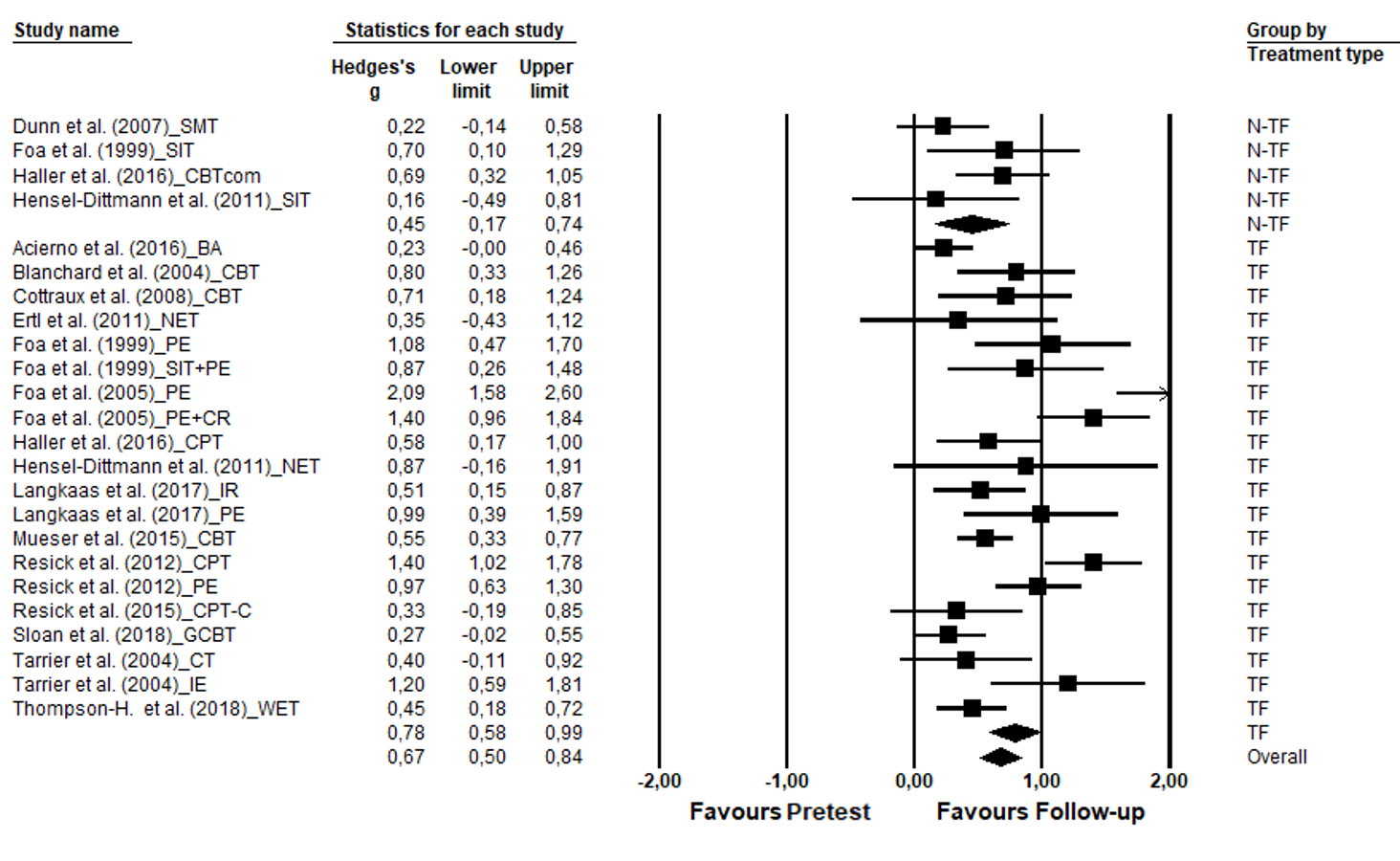


*Figure A6.* Within-group effect sizes (pretest – follow-up) of trauma-focused treatment (TFT, bottom) and non-TFT (top) for comorbid depressive symptoms.


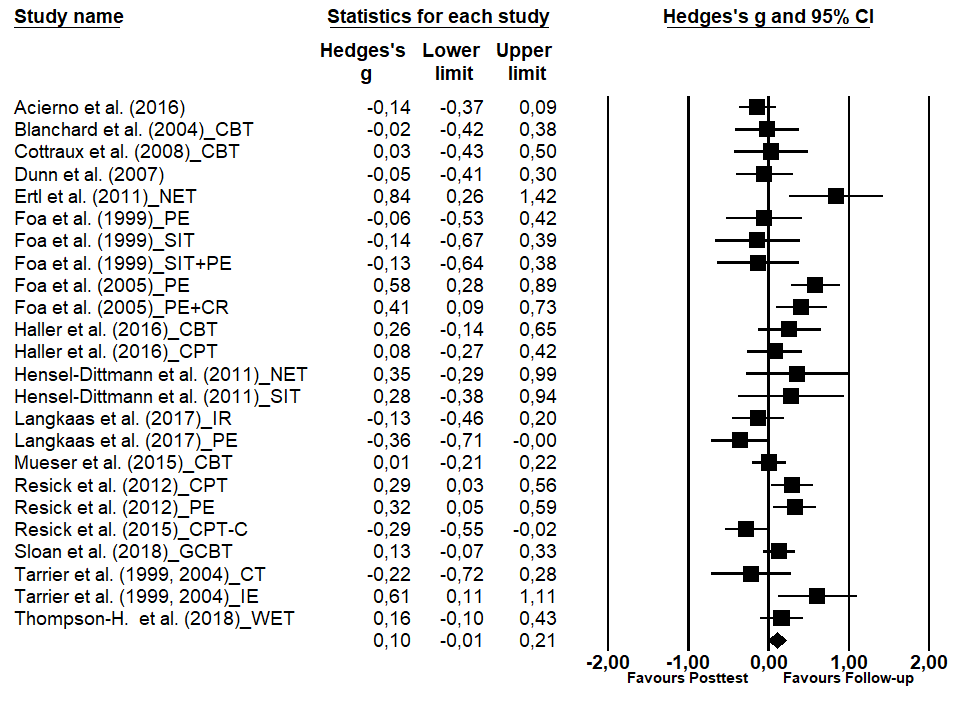

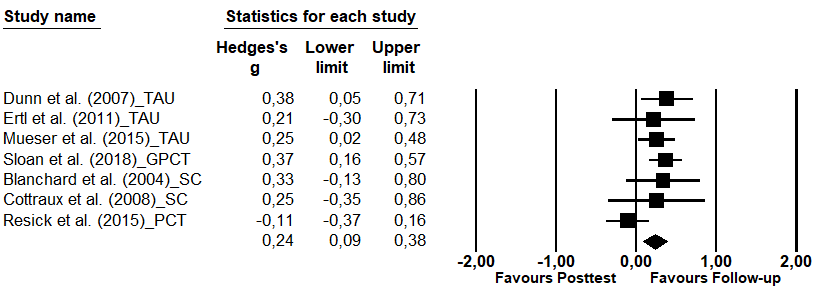


*Figure A7.* Within-group effect sizes (posttest – follow-up) of active treatments (top) and control conditions (bottom) for comorbid depressive symptoms.


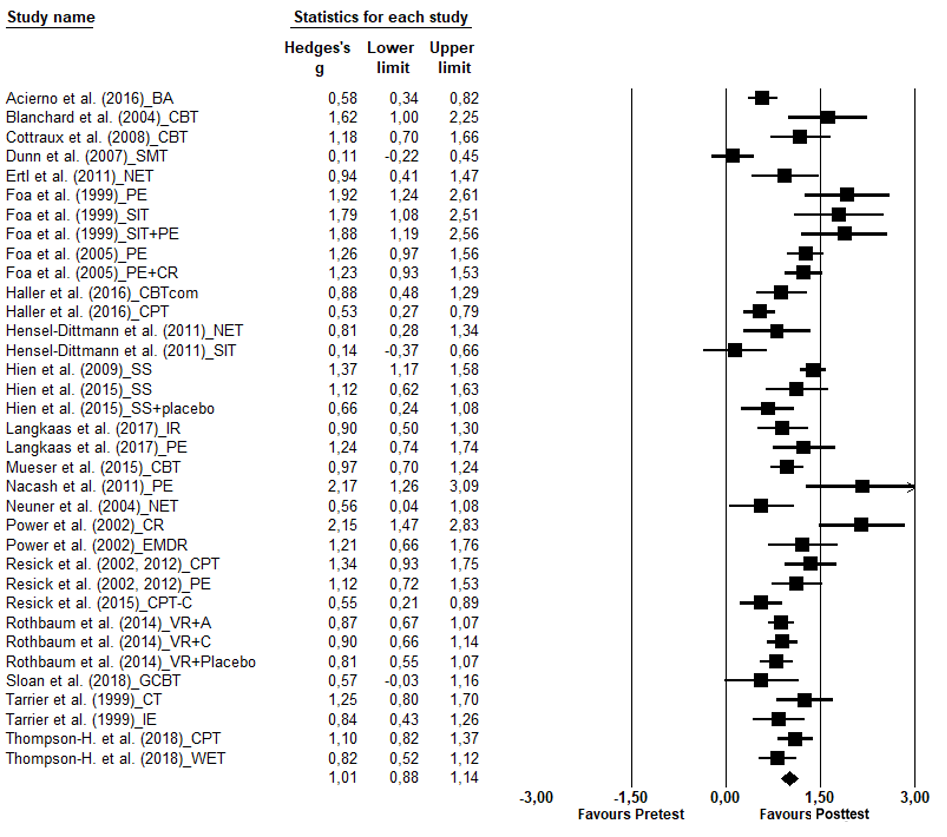


*
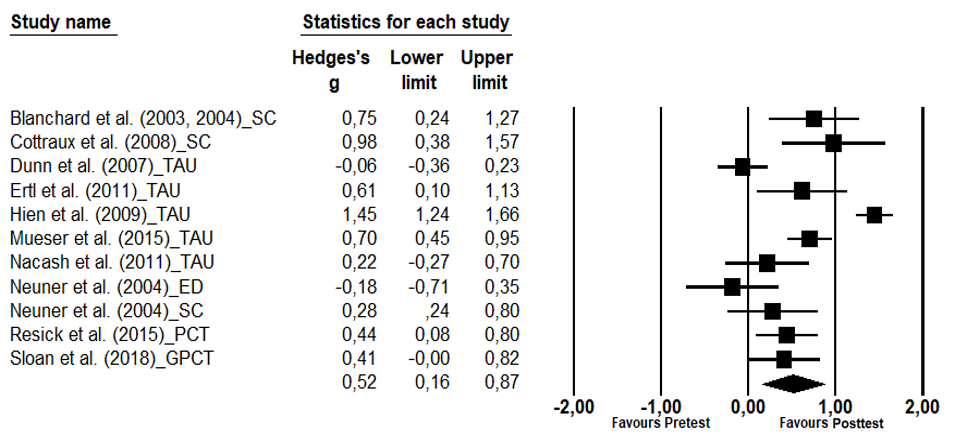
Figure A8.* Within-group effect sizes (pretest – posttest) for PTSD severity of active treatments (top) and control conditions (bottom).


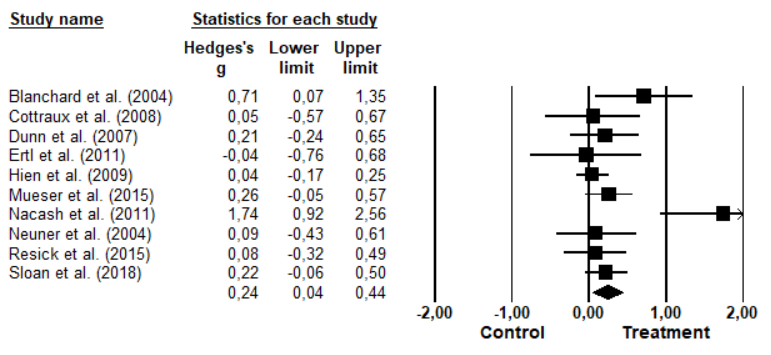


*
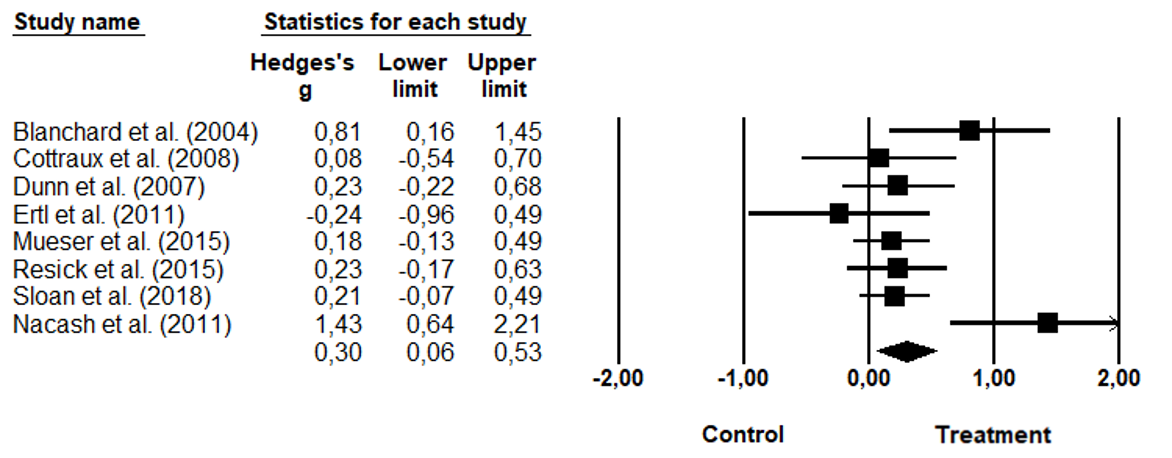
*

*Figure A9.* Between-group effect sizes for PTSD severity (top) and comorbid depressive symptoms (bottom) at posttest comparing active treatments with non-directive control groups.


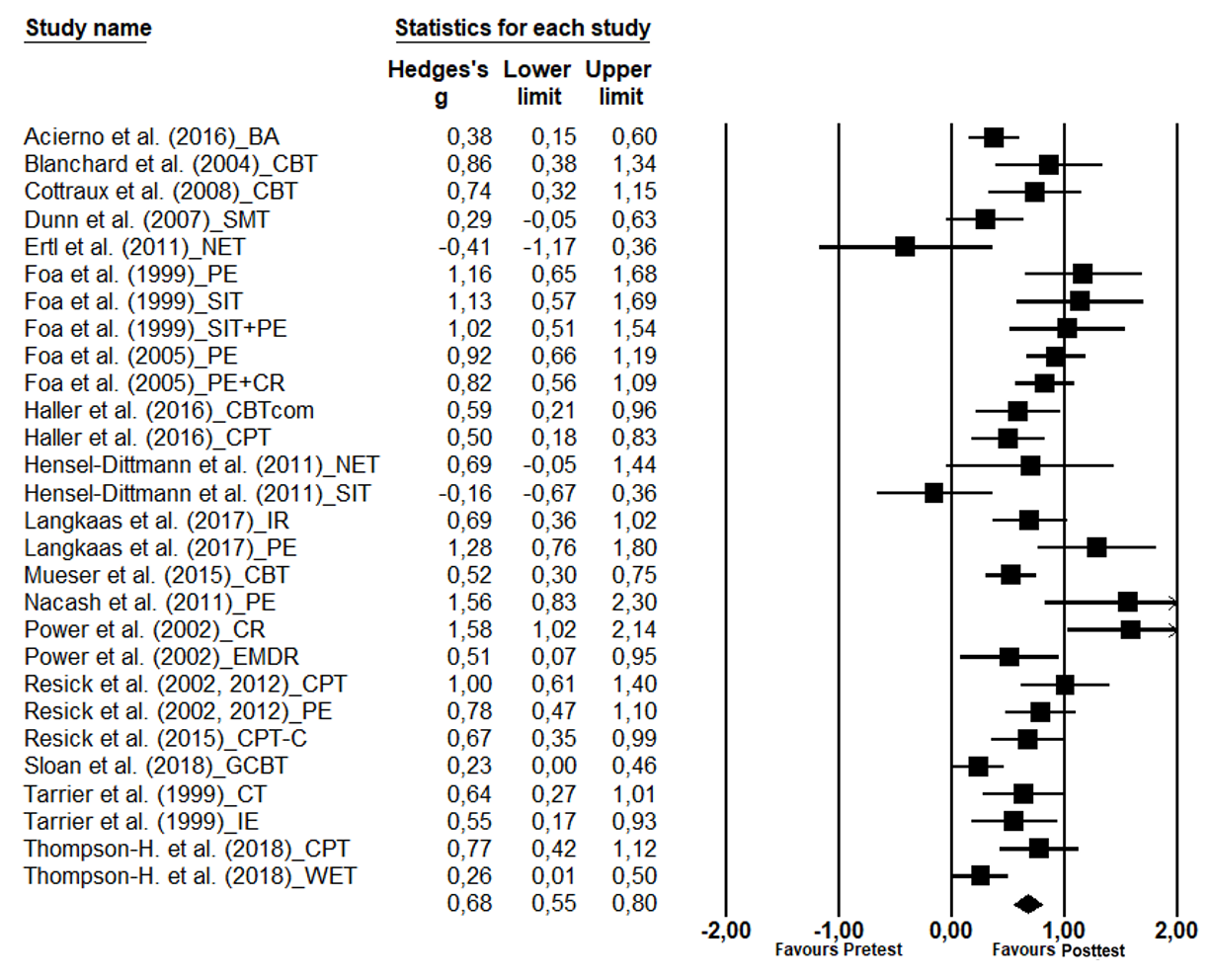


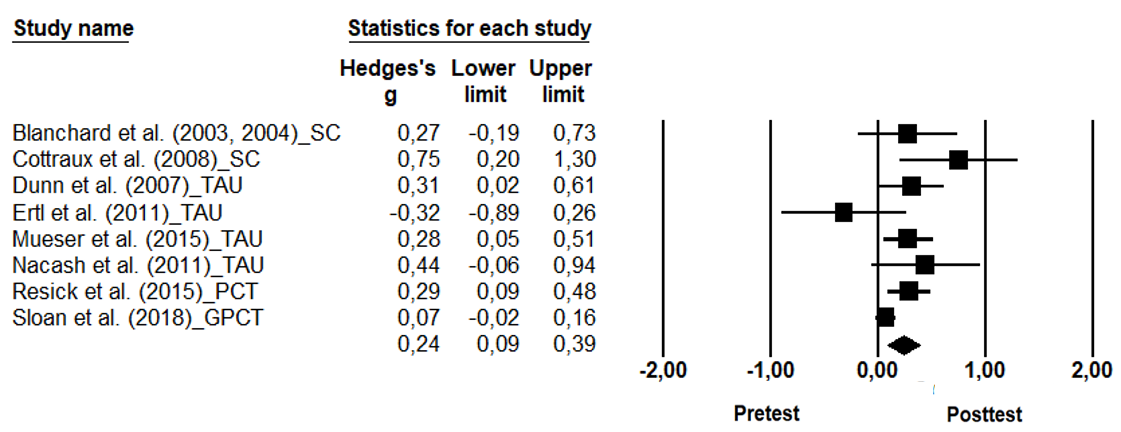


*Figure A10.* Within-group effect sizes (pretest – posttest) of active treatments (top) and control conditions (bottom) for comorbid depressive symptoms.
